# Worldwide prevalence of chagas cardiomyopathy—an analysis from the global burden of disease dataset

**DOI:** 10.1007/s15010-024-02408-5

**Published:** 2024-10-22

**Authors:** Abdul Mannan Khan Minhas, Rachel Marcus, Salim S. Virani, Michael D. Shapiro, Robert J. Mentz, Luis E. Echeverria, Jonathan T. Arcobello, Dmitry Abramov

**Affiliations:** 1 Department of Medicine, Baylor College of Medicine, Houston, TX USA; 2 Section of Cardiovascular Research, Baylor College of Medicine, Houston, TX USA; 3 Department of Cardiology, Medstar Washington Hospital Center, Washington, DC, USA; 4 Aga Khan University, Karachi, Pakistan; 5 Baylor College of Medicine, Texas Heart Institute, Houston, TX USA; 6 Cardiovascular Medicine, Wake Forest University School of Medicine, Winston Salem, NC USA; 7 Duke University Medical Center, Duke Clinical Research Institute, Durham, NC USA; 8 Heart Failure and Heart Transplant Clinic, Fundación Cardiovascular de Colombia, Floridablanca, Colombia; 9 Department of Infectious Diseases, Loma Linda University Medical Center, Loma Linda, CA USA; 10 Division of Cardiovascular Medicine, Loma Linda University Medical Center, 2068 Orange Tree Lane, Redlands, California 92374 USA

**Keywords:** Chagas disease, Chagas cardiomyopathy, Trypanosoma cruzi, T. cruzi

## Abstract

**Purpose:**

The worldwide prevalence of Chagas Cardiomyopathy (CCM) as well as the trends in the prevalence of CCM over time have not been well characterized.

**Methods:**

An analysis of the Global Burden of Disease (GBD) data from 1990 to 2019 was conducted to assess the burden of CCM. This study focused on determining the prevalence of CCM, along with its age-standardized prevalence rate (ASR) per 1,00,000 people, considering various patient demographics and geographical regions as defined in the GBD. Additionally, the study examined the temporal trends over this 30-year period by calculating the estimated annual percentage change (EAPC) in CCM prevalence for the global population and specific subgroups.

**Results:**

Worldwide, the GBD reported 220,166 individuals with CCM in 1990 and 2,83,236 individuals in 2019, with a decline in the ASR from 5.23 (3.34–7.47) to 3.42 (2.2–4.91) per 1,00,000 individuals during that period. In 2019, the prevalence was highest in individuals over age 70 and in males compared to females. Among available geographic classifications in 2019, Latin American regions had the highest rates (ASR of 39.49–61.15/1,00,000), while high income North American and Western European regions had the lowest rates (ASRs of 0.67 and 0.34/1,00,000, respectively). Between 1990 and 2019, the worldwide prevalence of CCM per 1,00,000 decreased (EAPC of −0.35, −0.37 to −0.32), with similar trends among most regions and subgroups.

**Conclusion:**

This analysis of the GBD data reveals both global and country-specific patterns in the prevalence and trends of CCM. Notably, CCM shows the highest prevalence in Latin American countries, although it’s also significantly present in regions beyond Latin America. Notably, the global age-standardized rate of CCM is on the decline, suggesting improvements in healthcare strategies or lifestyle changes across the world.

## Introduction

Chagas disease, caused by the protozoa Trypanosoma cruzi, is endemic in many Latin American countries but is also encountered outside of Latin America due to global migration [1, 2]. Chronic infection with Chagas disease may manifest with gastrointestinal and/or cardiac involvement characterized by arrhythmias, thromboembolic phenomena, and chronic Chagas cardiomyopathy (CCM). With chronic Chagas infection, the annual incidence of CCM is estimated at around 2% per year. [3] Over a 10-year period, the risk of experiencing a cardiac event, including CCM, is approximately 40%. Additionally, CCM is closely associated with high levels of morbidity and mortality [4]. Presentation can range from subclinical to symptomatic heart failure, including end-stage heart failure requiring advanced therapies or palliative care, to sudden cardiac death. Although the global burden of the full range of Chagas manifestations including mortality has been well characterized, [1] there are limited data on the global burden and disease trends specifically related to CCM. Therefore, we sought to assess the burden of CCM from the Global Burden of Disease (GBD) study, with a focus on both trends and regional differences as well as subgroup insights in disease prevalence.

## Methods

The data in this study were extracted from the GBD 2019 dataset using the research tools available at https://ghdx.healthdata.org/gbd-2019. GBD data were used to analyze the disease burden of heart failure due to Chagas disease between 1990 and 2019. Statistical modeling methods for estimating Chagas disease from the GBD, including data sources, have been previously described [1]. Specifically, the GBD estimated Chagas disease burden in endemic countries through country-level seroprevalence data with additional Bayesian data adjustment [1]. In non-endemic countries, the estimates were calculated by importation of prevalent cases by immigration [1]. Heart Failure within the GBD was likewise estimated using literature data, hospital data and claims data (modeled with spatiotemporal Gaussian process regression and DisMod–MR [a Bayesian metaregression method]), [5] with heart failure cause ultimately attributed to particular causes, including Chagas disease. [6]

The number of prevalent cases and their 95% uncertainty intervals (UIs) and age‐standardized prevalence rate (ASR) per 1,00,000 were determined by age, sex, year, socio-demographic index (SDI), regions, and countries and territories as characterized within the GBD. Age Standardized Rate is utilized in our analyses because it helps account for differences in the age distribution of the population across different groups and over time and is likewise indexed per 1,00,000 individuals so that prevalence estimates account for population growth and different population sizes of different groups.

The temporal trends from 1990 to 2019 were described using estimated annual percentage change (EAPC) and corresponding 95% confidence interval (95% CI) for global population and subgroups. Estimated Annual Percent Change, which approximates average annual percent change in ASRs during the study period, allows both an evaluation of trends over time as well as comparisons in the magnitude of change to the ASR among cohorts. For example an EAPC of -0.35 suggests that the ASR of that condition declines by approximately 0.35% per year over the course of the study period. EAPC was considered to be declining if the EAPC was negative and the 95% CI excluded 0.

## Results

In the GBD the total worldwide number of patients with Chagas Disease decreased between 1990 and 2019 from 7,292,889 to 6,469,285, with a corresponding decrease in ASR from 145.07 (126.74–165.59) to 79.86 (69.88–91.02).

During that period, the total number of CCM cases increased from 220,166 to 283,236 individuals, with a decline in the ASR from 5.23 (3.34–7.47) to 3.42 (2.2–4.91) per 1,00,000 individuals (Table 1). In 2019, the burden was highest in individuals over age 70 compared to younger individuals and in males compared to females. Among available geographic classifications in 2019 where CCM was noted, Latin American regions had the highest rates (ASR of 39.49–61.15/100,000), while high income North American and Western European regions had lower rates (ASRs of 0.67 and 0.34/100,000 respectively), (Table 1). The 2019 ASR for the United States was 0.71/100,000. Between 1990 and 2019, the worldwide prevalence of CCM decreased with an EAPC of -0.35 (-0.37 to -0.32), with similar trends seen among different age groups and between males and females. Declines in EAPC were also noted for Latin American regions and high income North American countries, while the EAPC increased in Western Europe and High-income Asia Pacific. The trends in ASRs and EAPCs of CCM during the study period mirrored those of Chagas Disease overall from the GBD, which saw ASRs decrease from 145.07 (126.74 to 165.59) to 79.86 (69.88 to 91.02), with an EAPC of -0.45 (−0.47 to −0.43). Among 204 countries studied in the GBD, the ASRs for the 51 countries with a detectable prevalence of CCM in 2019 are displayed in Table 2.

**Table 1:** Prevalence, Age Standardized Rates, and Estimated Annual Percent Change in Chagas Cardiomyopathy

	1990			2019			1990			2019			1990-2019		
	Number	95% UI		Number	95% UI		ASR	95% UI		ASR	95% UI		EAPC	95% CI	
Global	220166	139752	315866	283236	181832	407165	5.23	3.34	7.47	3.42	2.2	4.91	−0.35	−0.37	−0.32
Age groups															
25-49 years	82584	50994	122854	84705	52216	126847	4.86	3	7.23	3.12	1.92	4.67	−0.36	−0.38	−0.33
50-69 years	92358	59040	131759	127459	82264	183234	13.54	8.66	19.32	9.24	5.97	13.29	−0.32	−0.34	−0.29
70+ years	45224	30464	62006	71072	48034	97716	22.44	15.12	30.76	15.33	10.36	21.07	−0.32	−0.35	−0.29
Sex															
Male	142482	96950	194620	185477	126543	252930	7.15	4.91	9.68	4.67	3.2	6.35	−0.35	−0.37	−0.32
Female	77684	43701	123586	97759	55500	155060	3.56	2	5.65	2.27	1.29	3.6	−0.36	−0.39	−0.33
SDI															
High SDI	3149	2004	4521	3761	2422	5393	0.32	0.2	0.46	0.25	0.16	0.36	−0.23	−0.26	−0.19
High-middle SDI	89955	57869	128635	76580	49799	109055	8.23	5.31	11.76	3.87	2.51	5.52	−0.53	−0.55	−0.51
Middle SDI	68859	43323	99626	118561	75872	170684	6.16	3.93	8.86	4.64	2.99	6.64	−0.25	−0.27	−0.22
Low SDI	1486	939	2165	3075	1958	4526	0.58	0.37	0.84	0.56	0.36	0.81	−0.04	−0.12	0.06
Low-middle SDI	56711	35908	81969	81252	52212	116543	8.67	5.55	12.42	5.61	3.64	8.02	−0.35	−0.38	−0.32
Regions															
Endemic Regions															
Southern Latin America	71250	46178	101313	48812	32000	68694	153.25	99.36	217.85	61.15	40.05	86.16	−0.6	−0.62	−0.58
Andean Latin America	28915	18235	41644	32161	20744	46548	125.46	79.6	179.58	55.03	35.57	79.49	−0.56	−0.59	−0.54
Tropical Latin America	62080	39374	90263	100155	64247	145470	60.06	38.42	86.73	40.15	25.78	58.31	−0.33	−0.36	−0.3
Central Latin America	54182	33871	78296	96284	61396	137715	57.51	36.34	82.49	39.49	25.25	56.38	−0.31	−0.34	−0.29
Non-Endemic Regions															
High-income North America	2699	1717	3885	3396	2185	4873	0.84	0.53	1.21	0.67	0.43	0.96	−0.21	−0.24	−0.17
Western Europe	826	532	1172	2246	1444	3221	0.17	0.11	0.24	0.34	0.22	0.5	1.04	0.93	1.14
Australasia	116	74	164	65	42	94	0.52	0.33	0.73	0.16	0.1	0.23	−0.69	−0.71	−0.66
Caribbean	81	53	116	79	51	112	0.3	0.2	0.43	0.15	0.1	0.22	−0.49	−0.52	−0.45
High-income Asia Pacific	17	11	24	38	25	55	0.01	0.01	0.01	0.01	0.01	0.02	0.51	0.38	0.65

*UI* Uncertainty Interval, *CI* Confidence Interval, *ASR* Age standardized rate per 100,000 population, *EAPC* Estimated annual percent change, *SDI* socio-demographic index. Regional groups were defined based on Global Burden of Disease classification. Age groups are not age-standardized. Prevalence reported as 0 in 25–29 year age-group. Other geographic regions had no estimated prevalence of Chagas Cardiomyopathy (estimated prevalence of 0)

**Table 2:** Age standardized rates (ASR) of Prevalence per 1,00,000 Population for Chagas Cardiomyopathy in 2019

Country	ASR	95% IU	
Bolivia	185.65	118.3	267.95
Venezuela	77.95	50.67	108.51
Chile	65.94	43.32	92.72
Argentina	61.43	39.5	87.86
Mexico	45.13	28.69	64.86
Honduras	41.03	26.44	58.44
Brazil	40.48	26	58.83
Ecuador	32.56	20.73	47.16
Uruguay	32.21	20.33	45.81
Guatemala	31.81	20.44	46.03
Nicaragua	30.27	19.96	43.52
Peru	28.08	18.28	40.76
Paraguay	26.36	16.95	37.95
El Salvador	25.29	15.94	36.52
Panama	24.28	15.5	34.86
Costa Rica	21.12	13.22	30.36
Colombia	11.69	7.63	16.95
Spain	2.05	1.31	2.98
Belize	0.85	0.52	1.29
Grenada	0.76	0.34	1.41
Guyana	0.74	0.45	1.13
United States of America	0.71	0.45	1.02
Andorra	0.64	0.34	1.13
Puerto Rico	0.49	0.31	0.71
Israel	0.46	0.29	0.67
Italy	0.45	0.28	0.65
Suriname	0.4	0.24	0.62
Dominican Republic	0.38	0.24	0.55
Switzerland	0.37	0.23	0.54
Sweden	0.34	0.21	0.49
Canada	0.3	0.19	0.43
Portugal	0.22	0.14	0.31
Australia	0.19	0.12	0.28
Ireland	0.15	0.1	0.23
Antigua and Barbuda	0.1	0.05	0.21
Trinidad and Tobago	0.1	0.05	0.16
Netherlands	0.09	0.05	0.12
Denmark	0.08	0.05	0.12
Bahamas	0.07	0.03	0.12
France	0.06	0.04	0.09
Saint Lucia	0.05	0.01	0.14
Austria	0.05	0.03	0.07
Luxembourg	0.03	0.01	0.07
Iceland	0.03	0.01	0.08
Japan	0.02	0.01	0.03
Dominica	0.02	0	0.12
Germany	0.02	0.02	0.04
Cuba	0.01	0	0.01
Barbados	0.01	0.01	0.01
United Kingdom	0.01	0	0.01
Saint Vincent/Grenadines	0.01	0.01	0.02

## Discussion

Our results on the prevalence and trends of the global burden of CCM demonstrate several important findings. The prevalence of CCM is highest in endemic Chagas in Latin America, which have ASRs approximately 100 times higher than non-endemic regions, although the disease is also seen in North America, Europe, and other geographic locations worldwide. CCM is most prevalent in older individuals (over age 70) and more prevalent among males. Similar to global trends in the overall burden of Chagas disease, the burden of CCM is decreasing worldwide, although regional variability is noted. These results highlight the ongoing public health burden associated with CCM.

The current analysis expands on prior data about the prevalence of CCM. Prior to this analysis, there were limited data on worldwide prevalence and individual country prevalence outside of Latin America, including in the United States [2]. Additionally, there have been limited data regarding the trends in CCM. The current analysis confirms that CCM trends appear to mirror the previously reported declining prevalence of Chagas disease. The declining prevalence of Chagas disease has been attributed to multiple factors including improved screening of blood transfusion and vector control in endemic regions [7] with programs like the Southern Cone initiative and the Central American Initiative (IPCA), and initiatives to reduce maternal fetal transmission such as elimination of mother-to-child transmission (EMTCT) Plus. In addition, both the national and international level programs have been formalized to support diagnosis and treatment of individuals with Chagas disease, for example the Bolivian Chagas Platform and the National Chagas Program in Colombia.

The GBD prevalence data presented here need to be viewed in context of other prevalence estimates, including those that have described significantly higher prevalence of CCM in the US [8]. There may be various reasons for differences in prevalence estimates across studies and these differences may in part relate to underdiagnosis of CCM. Both acute Chagas infection and cardiomyopathy may be asymptomatic and CCM may be misdiagnosed as cardiomyopathy of alternate etiology, which may affect regional prevalence estimates. Underdiagnosis may occur because of healthcare system resource limitations or differences in what is perceived as cardiomyopathy, and these factors for underestimation may have geographic variation. The prevalence of CCM may also be underestimated because CCM may present with sudden cardiac death [2]. Additionally, prevalence data remain dependent on different statistical modeling approaches, which may explain differences among datasets.

We also highlight that CCM is more prevalent in older individuals and among males. The higher burden of both Chagas disease and CCM, as well as higher rates of mortality in males, [9] have been previously reported. These may be attributed to greater comorbidity burden in males or due to sex-specific effects of Chagas disease on the heart as noted by differences in myocardial damage noted on cardiovascular magnetic resonance imaging [10]. Regarding observations based on age, older individuals may be more likely to be diagnosed with CCM because they may be more likely to seek health care in certain regions or because age-associated comorbidities contribute to the risks of CCM [11]. Regarding regional trends, we note that CCM appears to be decreasing globally, although geographic variation exists. Western Europe and High incomes Asia Pacific countries did not demonstrate the declines in ASRs over time that was noted in other countries, which may be due to differences in transmission and migration patterns or greater focus on cardiomyopathy diagnosis among high-risk populations; although these trends need to be interpreted with caution due to low disease prevalence in these regions.

This analysis has limitations. This analysis does not evaluate the mortality associated with CCM but rather focuses on the prevalence of CCM itself with the goal of estimating the prevalence of this key consequence of Chagas Disease. It is notable that the prevalence estimates of Chagas Disease and, by extension, CCM from the GBD database demonstrate variations when compared to other sources. This discrepancy raises concerns about the potential underestimation of Chagas Disease and CCM cases in the GBD data, which might affect the perceived extent of these conditions. However, the strength of the GBD dataset lies in its consistent and reliable methodological approach [1] applied across a wide, global cohort over an extended period. This uniformity enhances the reliability and comparability of the data, contributing to a more accurate analysis of both prevalence and trends of these conditions across multiple countries. Therefore, despite its limitations, the GBD dataset remains a crucial resource for understanding the global patterns of Chagas Disease and CCM, offering significant insights for global health research and policymaking. Furthermore, it is important to collect consistent epidemiological data on understudied conditions such as Chagas Disease and CCM. Additional systemic public health efforts regarding Chagas Disease and CCM surveillance both in endemic regions and non-endemic regions will therefore be important to reduce collection bias and optimize future prevalence estimates.

In conclusion, this analysis from the GBD provides a comprehensive overview of the global and country-specific prevalence, along with trends, of CCM. CCM is most prevalent in Latin American countries, yet its presence is notable worldwide. Notably, the global prevalence of CCM is in decline, mirroring the trends observed in Chagas disease prevalence. These findings hold significant implications for public health initiatives. They underscore the necessity for targeted efforts to effectively characterize, prevent, and manage CCM on a global scale.

## Data Availability

The data are publicaly available.
